# Gut microbiota and the gut–brain axis in autism spectrum disorder and attention-deficit/hyperactivity disorder: A scoping review of shared and disorder-specific mechanistic pathways

**DOI:** 10.3934/microbiol.2026024

**Published:** 2026-08-25

**Authors:** Shifan Khanday, Afra Al Darmaki, Rokia Malahifci, Rima Alvavi, Sana Ahmed, Dina S. Nasr

**Affiliations:** Dubai Medical College for Girls, Dubai Medical University, Dubai 19099, United Arab Emirates

**Keywords:** gut–brain axis, gut microbiota, autism spectrum disorder, attention-deficit/hyperactivity disorder, neurodevelopmental disorders, scoping review, neuroinflammation, neurotransmitter

## Abstract

**Background:**

Autism spectrum disorder (ASD) and attention-deficit/hyperactivity disorder (ADHD) are among the most prevalent neurodevelopmental conditions worldwide, yet the biological mechanisms driving their onset and phenotypic variability remain incompletely understood. The gut–brain axis (GBA), a bidirectional network linking intestinal microbial communities to central nervous system function through endocrine, immune, neural, and metabolic pathways, has emerged as a candidate mechanism through which environmental factors interact with genetic susceptibility to shape neurodevelopmental outcomes.

**Objective:**

To systematically map the peer-reviewed evidence on gut microbiome-related mechanistic pathways in ASD and ADHD, we delineated features common to both disorders and identified areas of divergence and knowledge gaps requiring further investigation.

**Methods:**

A scoping review was performed following the PRISMA Extension for the Scoping Reviews (PRISMA-ScR) framework. PubMed, Scopus, and Google Scholar were searched for peer-reviewed studies published January 2018 through March 2025. Studies addressing gut microbiome composition, GBA signaling, or microbiome-directed interventions in ASD or ADHD were eligible for inclusion. Extracted data were synthesized using inductive thematic analysis via Covidence software.

**Results:**

Forty-five studies satisfied the eligibility criteria. Inductive analysis produced five mechanistic themes: Endocrine dysregulation and microbial metabolite signaling; neuronal signaling and neurotransmitter modulation; immune activation and neuroinflammation; gene–microbiota–environment interactions; and microbiota-targeted interventions. Studies on both disorders demonstrated modifications involving taxa with short-chain-fatty-acid-producing capacity; however, findings were varied and did not prove a common reduction in butyrate production. ASD was marked by pronounced dysregulation of the serotonergic system and tryptophan–kynurenine pathway and increased hypothalamic-pituitary-adrenal (HPA) axis responsiveness. On the other hand, the results in ADHD were majorly cross-sectional or preliminary, and the widely-m recognized dopaminergic dysfunction in ADHD has not been associated to specific microbiota alterations to date.

**Conclusions:**

Evidence demonstrates the GBA as a useful research conceptual for investigating ASD and ADHD, although the strength of mechanistic evidence differs substantially between the two disorders. Translating these insights into therapeutic strategies will require longitudinal, multi-omic research designs capable of establishing causality and enabling patient stratification for precision microbiome-based interventions.

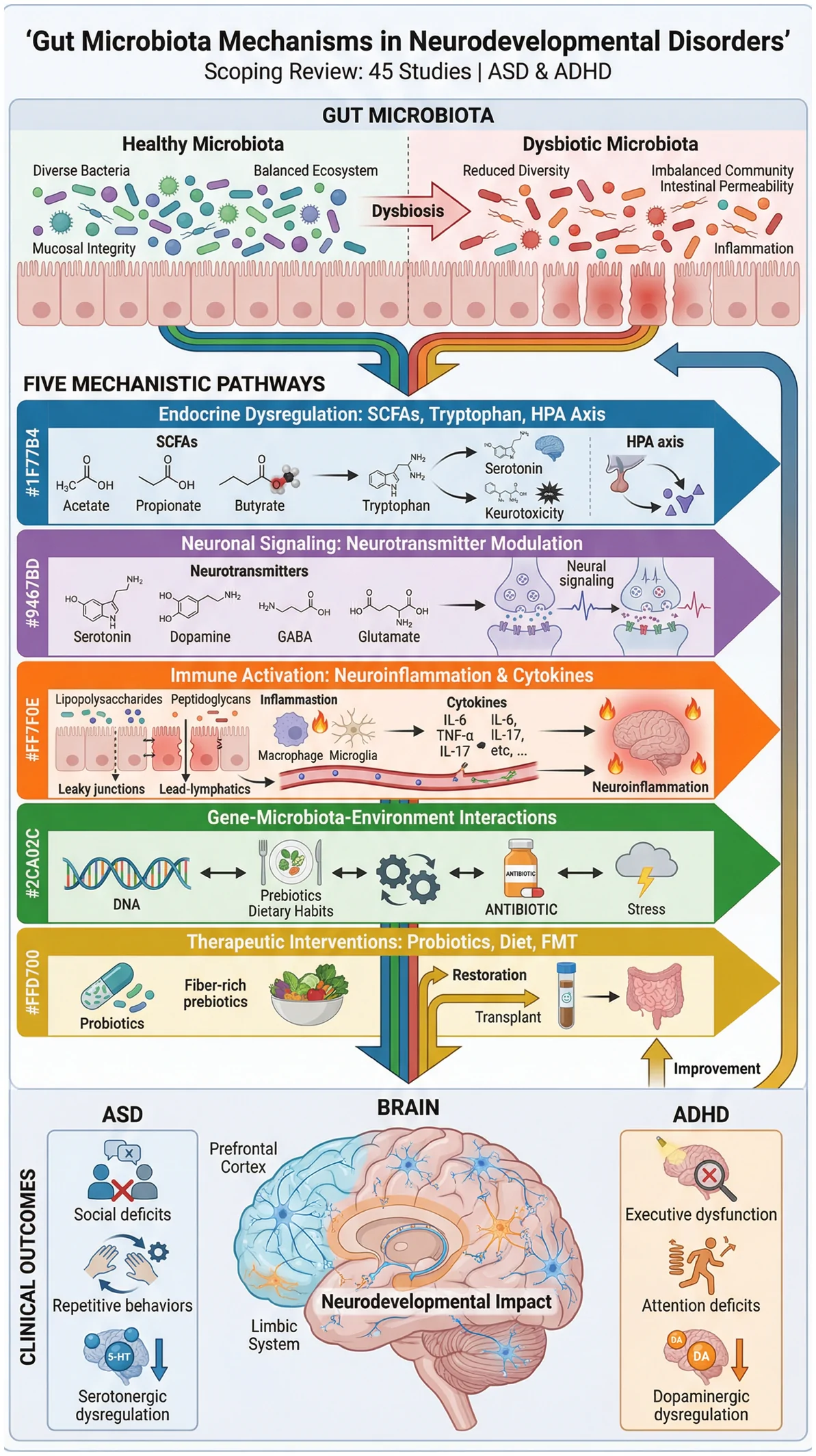

## 1. Introduction

Neurodevelopmental disorders (NDDs) impose a substantial global burden on affected individuals, families, and health systems. Autism spectrum disorder (ASD) and attention-deficit/hyperactivity disorder (ADHD) are among the most common and clinically recognized NDDs in childhood. ASD presents as a heterogeneous spectrum defined by impairments in social communication, restricted interests, and stereotyped behavior [1],[2]. ADHD, by contrast, is characterized by developmentally inappropriate levels of inattention, hyperactivity, and impulsivity that produce functional deficits across multiple life domains [3]. Despite their phenotypic differences, the two conditions co-occur at rates far exceeding chance, share overlapping neural substrates, and exhibit comparable heritability estimates, together suggesting a partially shared etiological architecture.

Genetic studies have made considerable progress in characterizing the molecular underpinnings of ASD. Whole-exome sequencing projects have catalogued over one hundred risk-associated variants implicated in synaptic scaffolding, neuronal migration, and signal transduction [4]; yet, genes alone account for only a fraction of observed phenotypic variance and cannot readily explain the steep secular rise in NDD diagnoses or the breadth of clinical heterogeneity within each diagnostic category. These observations have motivated growing interest in environmental modifiers that operate on or alongside genetic risk, particularly those active during prenatal and early postnatal neurodevelopmental windows.

The gut–brain axis has attracted particular attention as an environmentally sensitive biological mechanism linking intestinal physiology to CNS function. This axis encompasses vagal and enteric neural circuits, hypothalamic-pituitary-adrenal (HPA) endocrine signaling, humoral immune cascades, and the metabolic outputs of intestinal microbial communities [5]. The gut microbiome begins to assemble at birth and is profoundly shaped during the first years of life by mode of delivery, breastfeeding status, antibiotic exposure, dietary composition, and early psychosocial environment. Disruption of normal microbiome assembly during this sensitive period has the potential to produce lasting alterations in immune programming, neurotransmitter availability, and stress-axis calibration, all of which bear directly on neurodevelopmental trajectories.

Several researchers have identified variations of the gut microbiome in ASD. However, the relation of distinct taxa with ASD was variable across studies and populations [6]. ASD also has disorder-specific pre-clinical studies. For example, Sharon et al. [7] identified that transmission of gut microbiota from children with ASD or conventionally developed siblings to germ-free mice resulted in changes to ASD behavioral and neurological outcomes in mice. This does not demonstrate that gut microbiota causes ASD in humans, but instead microbiome-specific phenomena that specifically influence ASD in a mouse model.

On the other hand, in ADHD, some evidence from cross-sectional studies suggest variabilities in heterogeneity, taxonomic profile, and predicted functional ability of the gut microbiome [8]–[10]. However, there is no proof that these variabilities are causal for ADHD symptoms or associated with other known neurobiological alterations in ADHD. Therefore, findings characteristic to ASD, including Sharon et al. [7], cannot be generalized to ADHD.

**Figure 1.**
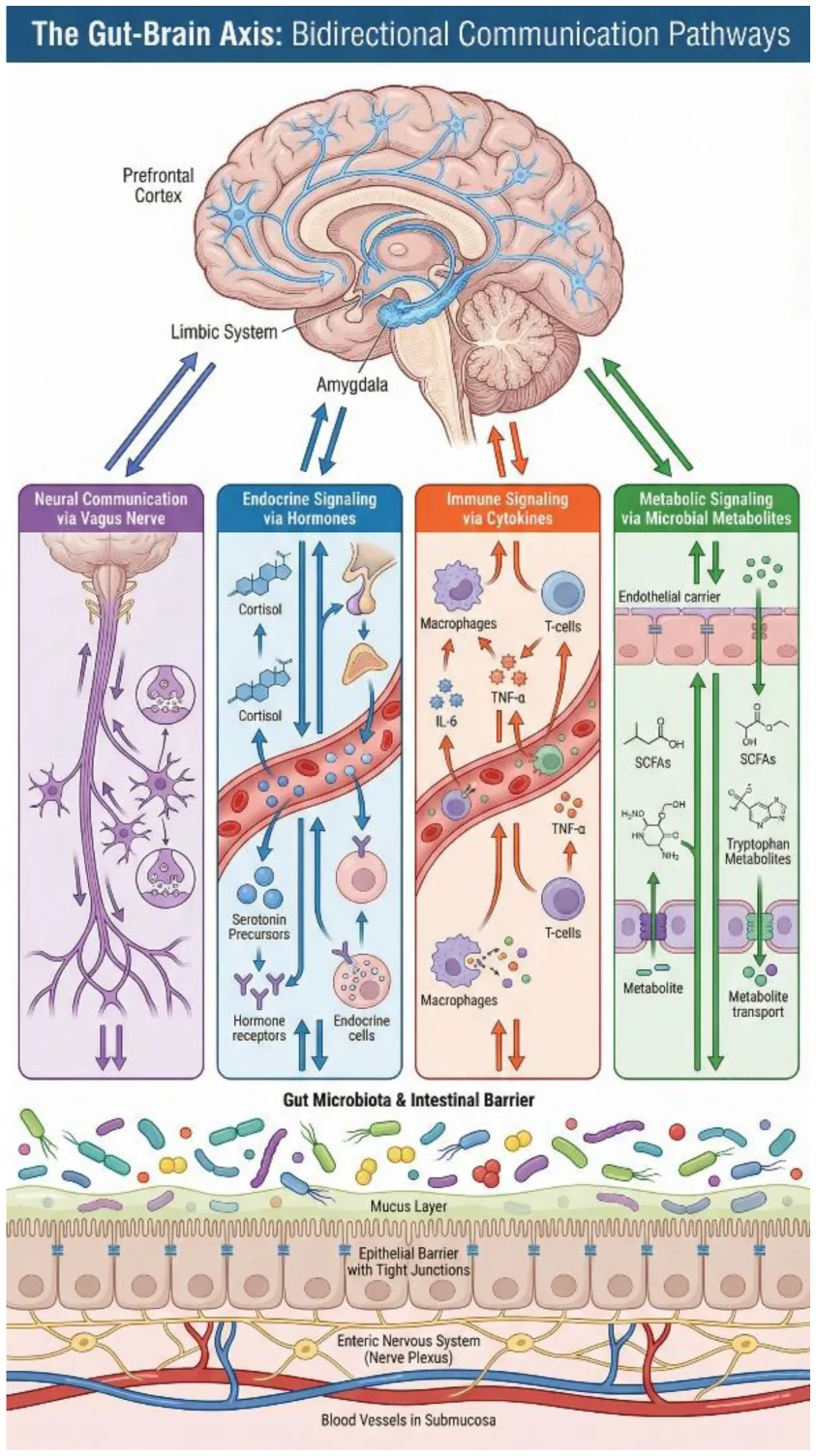
The gut–brain axis: Bidirectional communication pathways linking intestinal microbial communities to the central nervous system through neural, endocrine, immune, and metabolic signaling.

Despite this growing body of evidence, the mechanistic landscape remains fragmented. Individual studies tend to entail a single signaling pathway or a single diagnostic category, and methodological heterogeneity across the literature makes cross-study synthesis challenging. A scoping review with inductive thematic analysis is therefore an appropriate methodology to map the breadth of mechanistic evidence, identify convergent and divergent patterns across ASD and ADHD, and surface knowledge gaps warranting systematic investigation. Figure 1 represents a schematic illustration of the bidirectional communication between the gut and the brain. A schematic view of the potential mechanisms by which intestinal microbes may communicate with the CNS is provided in the diagram. The connections include neural, endocrine, immune, and metabolic routes and are realized through the vagal and enteric nervous system, the hypothalamic-pituitary-adrenal axis, inflammatory cytokines and intestinal barriers, and gut microbes' metabolites (short-chain fatty acids and tryptophan).

## 2. Materials and methods

### 2.1. Study design

This investigation was designed and conducted as a scoping review in accordance with the PRISMA Extension for Scoping Reviews (PRISMA-ScR) guidelines [11]. The scoping review framework was selected because our objective was to chart the mechanistic evidence landscape rather than to derive pooled quantitative estimates, and because the heterogeneity of study designs in this literature precludes formal meta-analysis. Covidence software was used throughout the screening and data extraction workflow.

**Figure 2.**
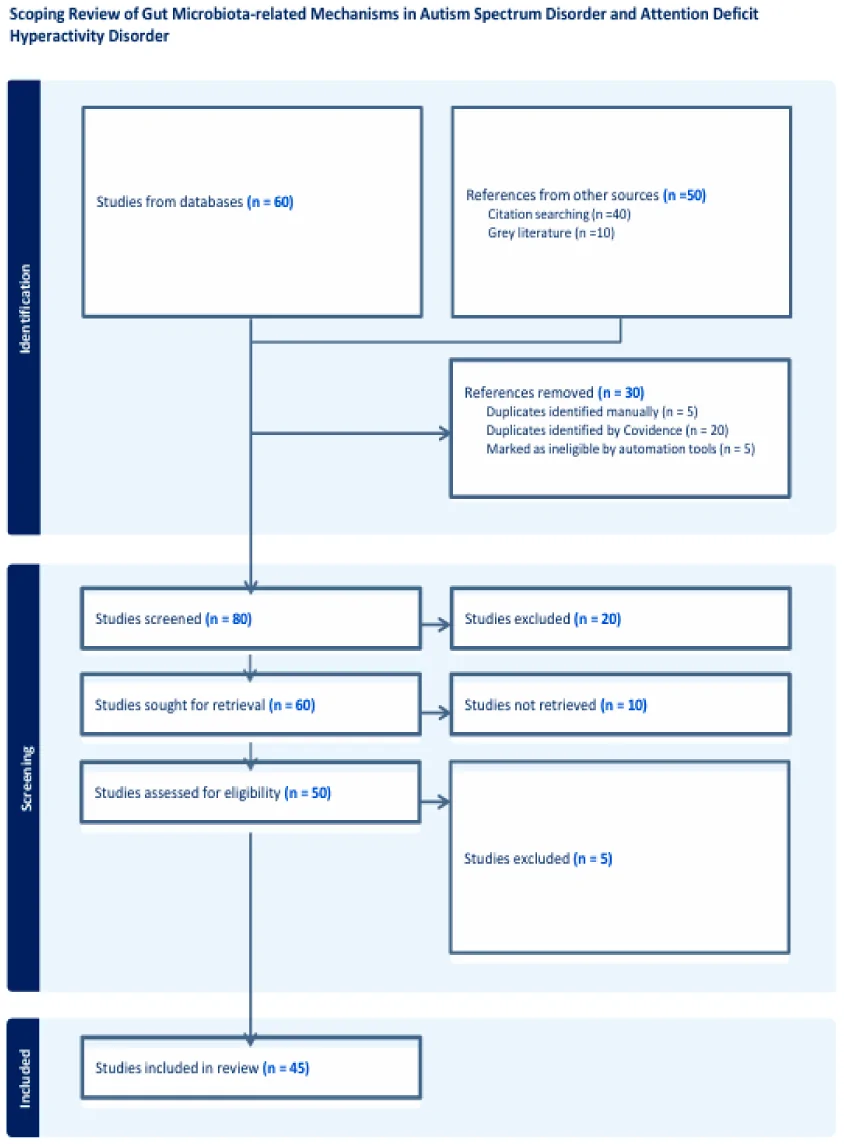
PRISMA-ScR flow diagram depicting study identification, screening, eligibility assessment, and final inclusion (n = 45 studies).

Figure 2 shows the PRISMA-ScR flow diagram for the study selection process. It indicates the step-by-step screening process involved in the identification of the research studies, including duplicates removal, initial screening of titles and abstracts, followed by a full-text eligibility screening. A total of 45 studies met all the eligibility criteria and were included in the scoping review to guide the thematic synthesis.

### 2.2. Search strategy

Systematic electronic searches were conducted in PubMed, Scopus, and Google Scholar, covering publications from January 2018 through March 2025. The search strategy combined the following terms using Boolean operators: “gut microbiota”, “gut microbiome”, “gut–brain axis”, “autism spectrum disorder”, “ASD”, “attention-deficit/hyperactivity disorder”, “ADHD”, “neurodevelopment”, “immune”, “endocrine”, “microglia”, and “short-chain fatty acids”. Reference lists of retrieved articles were hand-searched to identify additional eligible sources.

### 2.3. Eligibility criteria

Studies were included if they: (i) Were published in peer-reviewed journals in English; (ii) reported original data from human or animal populations, or represented systematic reviews or meta-analyses; and (iii) investigated a mechanistic relationship between gut microbiota or GBA signaling and ASD or ADHD. Studies were excluded if they were editorials, commentaries, or conference abstracts, or did not address gut–brain mechanistic pathways. No restriction was placed on study design, enabling inclusion of clinical trials, cohort studies, longitudinal studies, and preclinical experiments.

### 2.4. Data charting and thematic analysis

Title and abstract screening was performed independently by two reviewers, with disagreements resolved by discussion until consensus was achieved. Full-text review was conducted for all records passing the initial screen. For each eligible study, the following data were extracted: Study design, study population, microbiota findings, mechanistic pathways implicated, and primary outcomes. Extracted data were synthesized inductively, with recurring mechanistic themes identified iteratively through reviewer discussion. This process yielded five major mechanistic themes described in the Results and Discussion sections.

## 3. Results

Database searches returned a combined total of records from which, after duplicate removal, abstract screening, and full-text review, 45 studies met the eligibility criteria. The included studies spanned human observational and cohort designs, animal model experiments, and clinical intervention trials, and collectively addressed gut microbiota composition, GBA mechanistic pathways, and microbiome-directed therapeutic strategies. Thematic analysis produced five inter-related mechanistic themes, summarized in Table 1.

**Table 1.** Summary of the five mechanistic themes identified by thematic synthesis, including mechanistic content, disorder-specific evidence, and representative references.

Theme	Key mechanisms	ASD evidence	ADHD evidence	Key references
1. Endocrine dysregulation and microbial metabolites	SCFA imbalance; tryptophan–kynurenine shift; HPA axis dysregulation; enteroendocrine disruption	Reduced butyrate taxa; serotonin–kynurenine diversion; quinolinic acid accumulation; HPA hypersensitivity	Microbiome compositional and predicted-functional differences; direct microbiome–HPA-axis or cortisol connections remain scarce	[6],[8]–[10],[12],[13]
2. Neuronal signaling and neurotransmitter modulation	Microbial regulation of serotonin, dopamine, GABA, glutamate; synaptic gene expression; E/I imbalance	Serotonergic disruption; ASD microbiota transfer induces social deficits in germ-free rodents	Microbiome associations connected to taxonomy and predicted function; known frontostriatal alterations have insufficient mechanistic connection to certain microbial changes	[8],[9],[14]
3. Immune activation and neuroinflammation	Intestinal hyperpermeability; LPS translocation; IL-6, TNF-α, IL-17 elevation; microglial priming; aberrant synaptic pruning	Altered inflammatory signaling and microglial outcomes noted in selected studies; gut–immune genetic effects still remain model-specific	Low-grade systemic inflammation; cytokine burden correlates with symptom severity	[15]–[18]
4. Gene–microbiota–environment interactions	Host genetic differences may engage with gut microbiota and environmental influences; available evidence persists gene- and model-specific.	Model-specific studies of gene–microbiota associations, including results from Shank3 knockout mice; broader effects across ASD-associated genes remain unproven.	Evidence remains constrained; direct gene–microbiota mechanisms influencing dopaminergic function have not been proven	[19]
5. Microbiota-targeted interventions	Probiotics, prebiotics, dietary modification, FMT; outcomes vary by strain, age, and baseline microbiome	Modest GI and behavioral gains in probiotic/FMT trials; animal studies demonstrate social-deficit reversal	Preliminary evidence of improved behavioral regulation; heterogeneous findings; limited RCT data	[20]–[22]

### 3.1. Theme 1: Endocrine dysregulation and microbial metabolites

Evidence linking gut microbiota to endocrine dysfunction was the most frequently reported and mechanistically detailed theme. Central to this theme was SCFA production, principally acetate, propionate, and butyrate, by anaerobic intestinal bacteria. Butyrate maintains blood–brain barrier (BBB) integrity via tight junction reinforcement, guides microglial maturation in the developing brain, and exerts epigenetic effects through histone deacetylase inhibition, thereby influencing neuronal gene expression and synaptic plasticity [12]. The modifications in taxa with short-chain-fatty-acid-producing capacity demonstrated in ASD and ADHD were not uniform between the studies; thus, these taxonomic findings do not detect a shared decrease in butyrate production.

In ASD, tryptophan catabolism emerged as a second major endocrine-microbial intersection. Gut dysbiosis is associated with shifts in tryptophan catabolism, with evidence of increased relative flux through the kynurenine pathway. It is important to note that approximately 95% of dietary tryptophan is metabolized through the kynurenine pathway under physiological conditions, with only 1–2% directed toward serotonin synthesis; dysbiosis modulates the relative balance of these concurrently active pathways rather than diverting tryptophan from a default serotonergic route. Furthermore, central serotonin synthesis depends primarily on tryptophan transport across the blood–brain barrier via the large neutral amino acid transporter (LAT1), not on peripheral serotonin availability per se, as peripheral 5-HT is largely excluded from the CNS. Elevated kynurenine pathway activity in ASD may also reflect systemic inflammation, through cytokine-mediated induction of IDO1, rather than direct microbial enzymatic conversion. Within the kynurenine pathway, quinolinic acid is an NMDA receptor agonist capable of excitotoxic effects at pathological concentrations; at physiological concentrations, its primary role is as an NAD+ biosynthesis intermediate, and its effects should therefore be understood as concentration-dependent [6].

HPA axis dysregulation constituted a third endocrine mechanism. Germ-free rodent models display exaggerated corticosterone responses to stress, partly corrected by specific microbial colonization; Sudo et al. [13] explicitly reported partial rather than full normalization of the HPA response, a distinction with meaningful clinical implications regarding residual vulnerability. Notably, reconstitution in that study was performed at 6–16 weeks of age (1–3 weeks prior to testing), not during the neonatal period, meaning the observed sensitive-period effect reflects procedural timing rather than developmental programming during infancy. The translational relevance of this time-dependent microbiome effect to human HPA axis maturation remains unestablished, and the term “programming” should be understood as indicative of a sensitive period rather than proven irreversibility [13]. In ADHD, microbiome studies have demonstrated compositional and predicted-functional variation, but direct findings connecting particular microbial alterations to HPA-axis or cortisol dysregulation remains limited.

Figure 3 presents a schematic synthesis of the five key mechanistic themes identified through thematic synthesis in this review: Endocrine dysfunction and microbial metabolite signalling; neuronal signalling and neurotransmitter function; immune activation and neuroinflammation; gene–microbiota–environment interactions; and microbiota-targeted interventions. This schematic highlights how these putative mechanisms might interact within the gut–brain axis to contribute to ASD and ADHD pathophysiology. However, it is important to note that the representation of mechanisms in this figure is not necessarily indicative of their relative involvement in the aetiopathogenesis of the two neurodevelopmental conditions.

**Figure 3.**
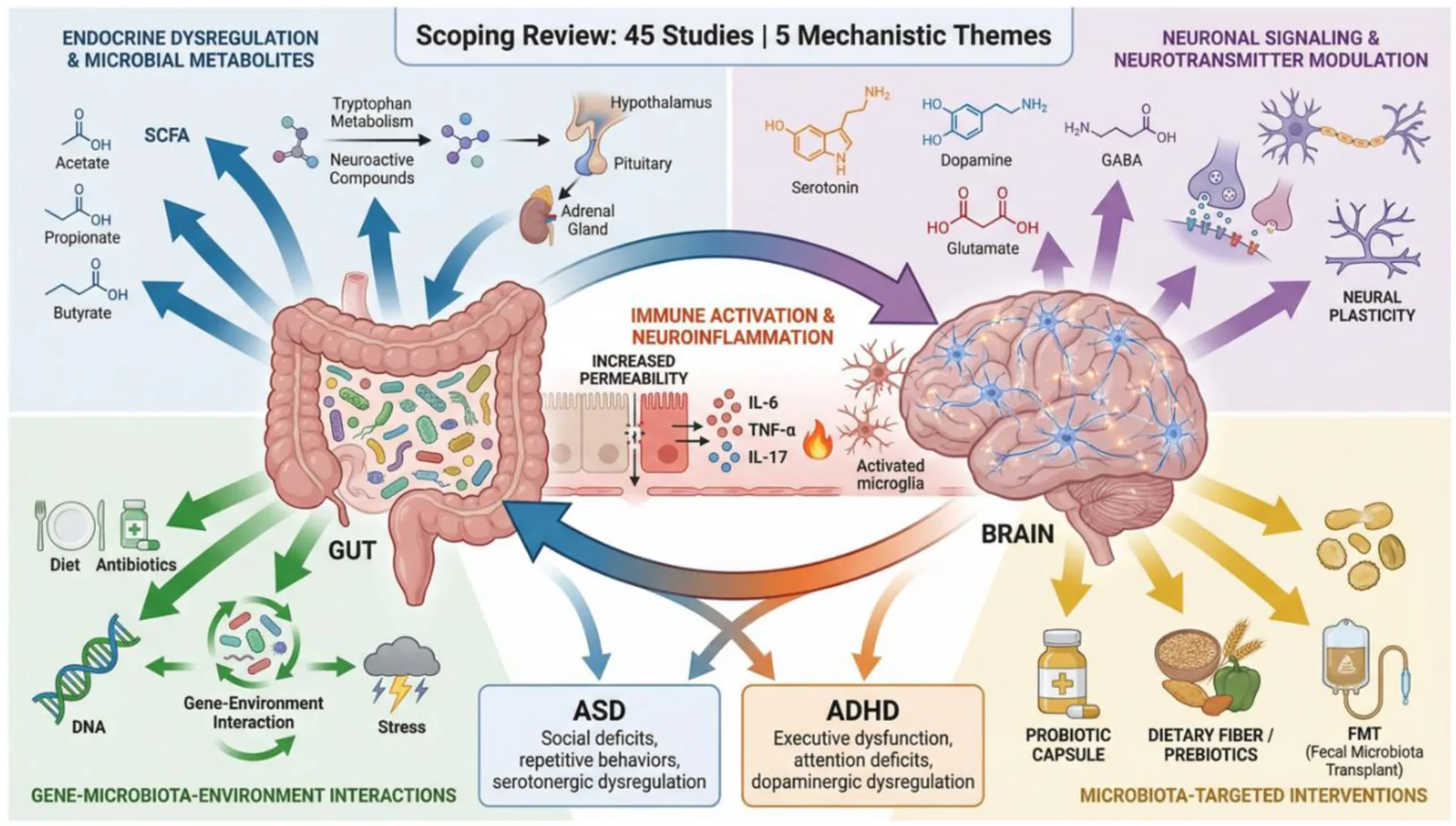
Overview of the five mechanistic themes linking gut microbiota dysbiosis to ASD and ADHD: Endocrine dysregulation, neuronal signaling, immune activation, gene–environment interactions, and microbiota-targeted interventions, and their respective clinical outcomes. The pathways illustrated are conceptual and do not imply equivalent causal or mechanistic evidence in ASD and ADHD.

### 3.2. Theme 2: Neuronal signaling, neurotransmission, and synaptic plasticity

A broad body of evidence demonstrated that gut microbial communities regulate the central availability of key neurotransmitters by producing neuroactive molecules and modifying biosynthetic precursor availability. Microbial species produce or modulate precursors of serotonin, dopamine, GABA, and glutamate, each integral to attention, executive function, emotional regulation, and social cognition [5],[23].

In ASD, the mechanistic primacy of neuronal disruption was demonstrated by colonization experiments: Transfer of human ASD-derived microbiota into germ-free mice reproducibly induced reduced sociability, increased repetitive behavior, and widespread changes in synaptic gene expression [7]. Translational evidence was provided by a double-blind randomized trial of *Lactobacillus plantarum* PS128, which produced measurable improvements in social responsiveness and anxiety in children with ASD [21].

In ADHD, microbiome studies describe the relations between gut microbiota and inferred microbial function and ADHD; for instance, Jiang et al. [8] demonstrated variations in taxonomic profile in previously untreated children with ADHD, while Aarts et al. [10] demonstrated a preliminary relation between predicted microbial function and neural activity to reward expectancy. Significantly, frontostriatal and dopaminergic alterations are part of the endophenotype of ADHD [14]. Regardless of the presence of both bodies of evidence, there is no empirical evidence on the role of particular bacteria in the pathophysiology of dopamine dysregulation. In essence, a microbiota–dopamine axis in ADHD is postulated but not demonstrated. Figure 4 represents the dysbiosis-neuroinflammation hypothesis, which suggests a mechanism by which the intestinal microbiota alterations can affect the central nervous system. The process starts with dysbiosis leading to increased intestinal permeability resulting in lipopolysaccharide translocation that can cause peripheral immune activation. Then, pro-inflammatory cytokines from peripheral sources can trigger microglial activation, which can lead to synaptic pruning and dysfunction. It is noteworthy that this figure illustrates only one possible mechanism of interaction between microbiota and the host, and there is no direct evidence for ASD or ADHD causation.

**Figure 4.**
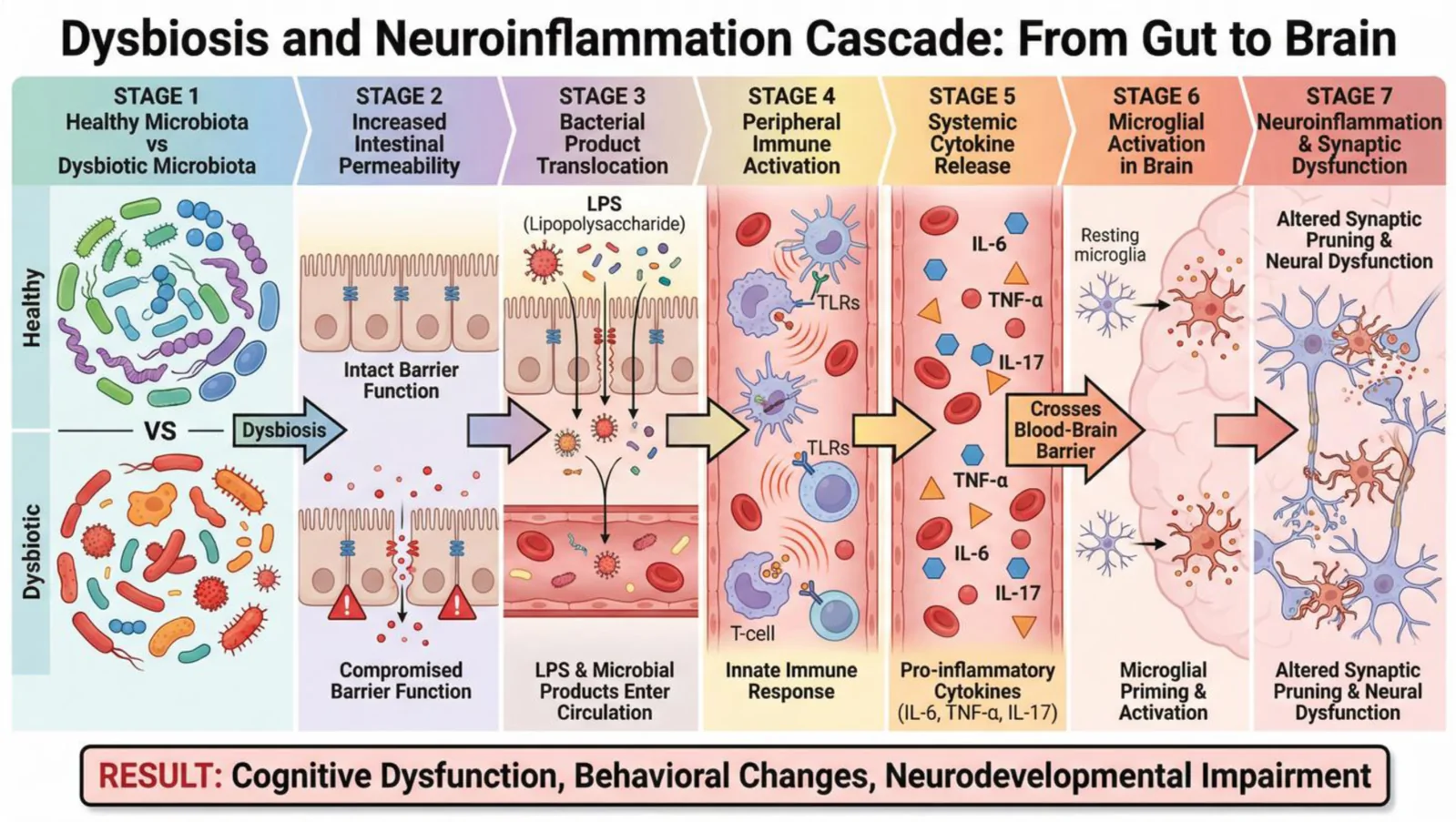
Dysbiosis and neuroinflammation cascade: Seven sequential stages from healthy versus dysbiotic microbiota through increased intestinal permeability, LPS translocation, peripheral immune activation, systemic cytokine release, microglial priming, and neuroinflammation with synaptic dysfunction.

### 3.3. Theme 3: Immune activation and neuroinflammation

Immune dysregulation emerged as the third dominant mechanistic theme. Neurodevelopmental studies have shown the role of altered intestinal barrier function and peripheral inflammatory pathways. Nonetheless, findings on the relationship between intestinal microbiota and neurodevelopmental outcomes were varied, while evidence for the involvement of peripheral inflammation in ADHD pathophysiology was more uniform. In ADHD, a connection has been demonstrated between inflammatory cytokines and clinical or neuropsychological features [17],[18]. Significantly, none of these studies showed that gut microbiota dysbiosis may be causally involved in increased levels of cytokines, and, thereafter, in the pathophysiology of ADHD through a microbiota–LPS–inflammation axis. In a mouse model of maternal immune activation, Hsiao et al. [24] demonstrated alterations in intestinal permeability and patterns of microbes and metabolites, indicating that treatments targeted at the microbiota modified specific gastrointestinal and behavioral issues.

At the CNS level, peripheral immune activation leads to microglial priming. Microglia, the brain's resident immune effectors, perform essential synaptic pruning during critical postnatal developmental periods [15]. Dysbiosis-induced inflammation may prime microglia toward hyperactivation, impairing synaptic pruning precision and producing aberrant circuit formation. Post-mortem neuropathological evidence from individuals with ASD demonstrates altered microglial morphology and elevated density in prefrontal and cerebellar tissue [15],[16].

Genetic architecture further intersects with immune pathways. Genetic influences to gut and immune phenotypes in ASD are multifaceted and possibly gene- and model-targeted, as reflected by the inconsistent effects on intestinal barrier function or peripheral cytokine profiles across different studies. The concept that variants in SHANK3, PTEN, and MECP2 uniformly disrupt intestinal epithelial functionality or modulate peripheral cytokine responses is therefore not backed by the presented evidence.

### 3.4. Theme 4: Gene–microbiota–environment interactions

A subset of studies entailed how genetic variation and environmental exposures jointly influence gut microbiome composition and neurodevelopmental outcomes. Evidence on gene-microbiota relation in the background of ASD is limited and model-specific. Thus, in Shank3 knockout mice, microbiota dysbiosis and behavioral and neurochemical abnormalities were noted after probiotic treatment, indicating a relation between this ASD-associated gene and the gut microbiota [19]. However, generalizing these results to PTEN, MECP2, or other ASD-related genes would be incorrect in the absence of similar investigations in the corresponding genetic model.

Environmental determinants of early microbiome assembly, including mode of delivery, breastfeeding duration, antibiotic prescriptions, and dietary composition, were identified as modulators capable of amplifying the neurodevelopmental consequences of genetic risk. The gene-microbiota studies in ADHD is scarce and direct evidence that such connections are causal is missing, especially with respect to the concept that microbiota-induced perturbations in dopaminergic function can occur in a genetically predisposed brain.

### 3.5. Theme 5: Microbiota-targeted interventions

The fifth theme encompassed clinical and preclinical evidence for therapeutic modifiability of the microbiome–GBA–NDD pathway. In ASD, an open-label microbiota transfer therapy study demonstrated enhancements in gastrointestinal and ASD-related symptoms [20]. A randomized controlled trial of *Lactobacillus plantarum* PS128 demonstrated statistically significant reductions in emotional reactivity and social withdrawal subscale scores versus placebo [21]. Animal studies provided mechanistic support: Restoration of commensal microbial populations in offspring of high-fat-diet-fed mothers reversed social behavioral deficits and corrected synaptic plasticity deficits in the mesolimbic circuit [22].

Evidence for microbiota-targeted interventions in ADHD remains at a very early phase, and the evidence base is scarce to claim the conclusions regarding efficacy for core ADHD symptoms. Across both disorders, response variability was high and appeared to depend on the strain administered, baseline microbiome composition, and age at intervention. Figure 5 shows the main microbiota-targeted approaches explored for the modulation of gut–brain axis in ASD and ADHD, including the use of probiotics, prebiotics, dietary interventions, and fecal microbiota transplantation. The rationale for each approach is based on possible beneficial effects on microbiota composition and/or metabolites, intestinal barrier function, and immune and neuroactive pathways. A limited number of clinical and preclinical studies are available, providing inconsistent evidence on the possible gastrointestinal, behavioral, and psychiatric outcomes of microbiota-targeted interventions in ASD and, more limited, in ADHD.

**Figure 5.**
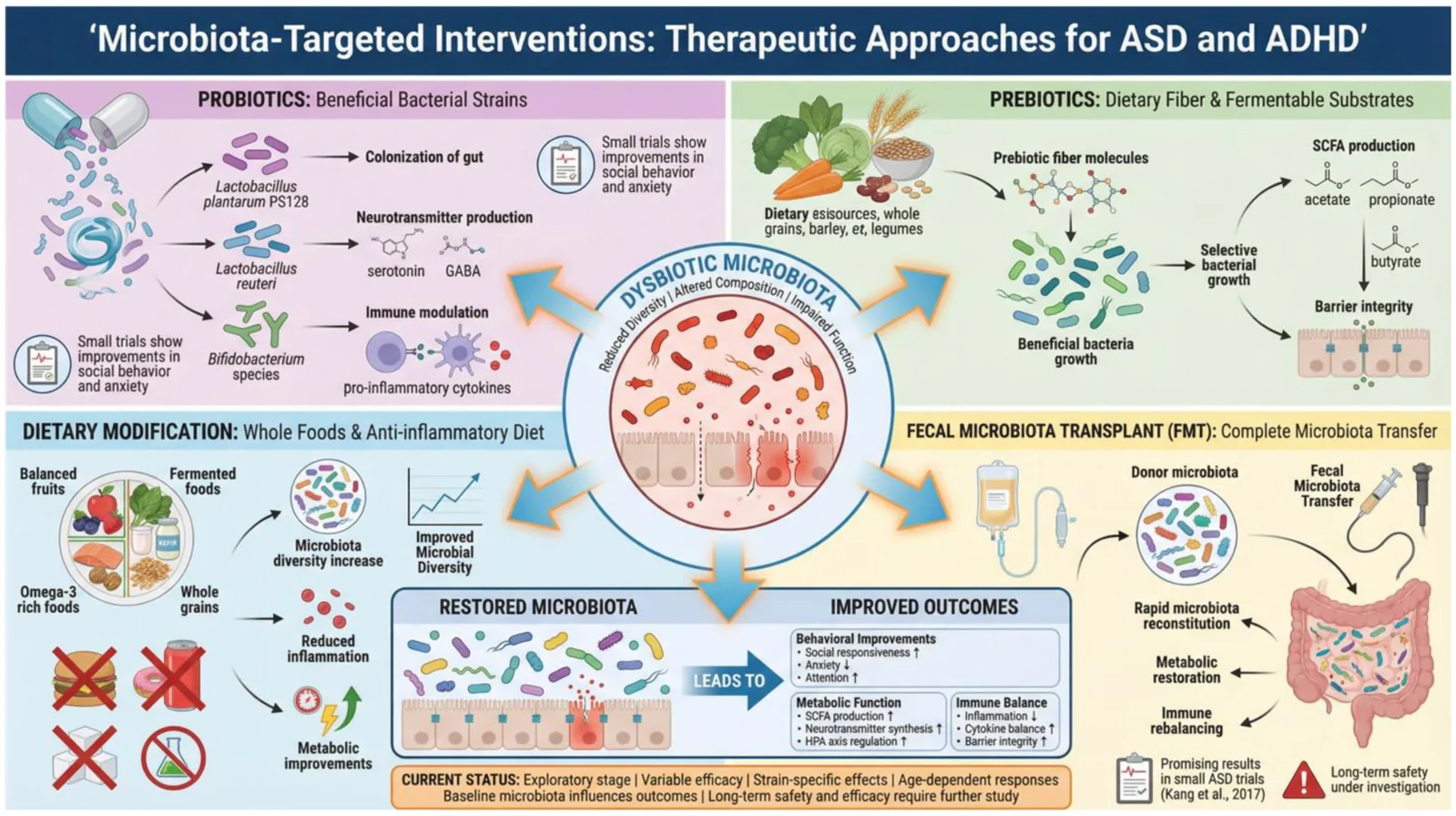
Microbiota-targeted intervention strategies for ASD and ADHD: Mechanisms and clinical evidence for probiotics, prebiotics, dietary modification, and fecal microbiota transplantation (FMT).

## 4. Discussion

The five themes stemming from this scoping review emphasize the gut microbiome and the gut–brain axis as areas of possible research in ASD and ADHD, but the evidence base is markedly different between the two conditions. ASD comprises both preclinical mechanistic findings, while the evidence for ADHD is largely correlational. Endocrine, neuronal, immune, genetic, and environmental components of the GBA interact in a dynamic network whose perturbation at any node has the potential to propagate disruption across the system. This network model is broadly consistent with the multisystem clinical phenotypes of both disorders and with the observation that neither condition can be adequately explained by genetic architecture alone.

### 4.1. Shared and divergent microbial signatures

Some studies on ASD and ADHD have shown dysbiosis consisting of short-chain-fatty-acid-producing bacteria at the genus level [6],[8],[9]. Nevertheless, in each instance, specific genera were noted to be changed, and increases or decreases were not uniformly observed with the concept that butyrate production is reduced. The results therefore demonstrate a potential common pattern in the modification of SCFA production capacity, but without portraying a common deficit especially in butyrate production.

In contrast, the evidence base differs notably between the two conditions. For ASD, there are preclinical finding in which experimental ASD is partly reversed through experimental manipulation of the gut microbiota [7]. For ADHD, although frontostriatal dopaminergic dysfunction is proven at the neurobiological level [14], the published microbiome researches mainly demonstrates connections at the taxonomical or predicted functional profile levels [8],[10]. The relationship between the two is currently theoretical, as there have been no researches portraying a direct mechanistic link. Indeed, it is plausible that metabolites, inflammatory cytokines, or precursor availability from the microbiota could influence dopaminergic function; however, a microbiota–dopamine concept for ADHD has yet to be demonstrated.

### 4.2. Endocrine-microbial mechanisms: Serotonin, butyrate, and HPA axes

The tryptophan–kynurenine shift documented in ASD merits particular attention because serotonin exerts organizing effects on developing cortical circuits that are unique to early postnatal life. During the first years of brain development, serotonin acts as a trophic factor supporting dendritic arborization, synaptogenesis, and thalamocortical connection formation [25]. Microbiota-driven alterations in tryptophan catabolism during this window may therefore carry developmental significance beyond effects on peripheral serotonin, given the additional roles of kynurenine-pathway metabolites in neuroinflammation and excitotoxicity.

Experimental research shows that microbial colonization can affect HPA-axis reactivity [13], offering a general biological setup for microbiome–stress interplay; however, disorder-specific evidence proving this mechanism in ASD or ADHD remains scarce. The HPA axis is a regulator of immune function and BBB permeability; chronic HPA dysregulation may therefore amplify the neuroimmune consequences of primary microbiome perturbation, creating a self-reinforcing cycle of endocrine and immune dysregulation.

### 4.3. Neuroinflammation and the microglial nexus

The convergence of intestinal hyperpermeability, systemic cytokine elevation, and central microglial activation positions the neuroimmune axis as a critical mediator between peripheral gut dysbiosis and central neurodevelopmental disruption. The microglial priming state induced by LPS and inflammatory cytokines is particularly consequential during the early postnatal critical period of synaptic pruning, when excitatory synapse density is sculpted from an overproduced baseline toward the refined connectivity of the mature brain [15]. Microglial hyperactivation during this window may produce excessive or aberrant pruning in circuits governing social behavior in ASD or executive control in ADHD, yielding circuit-level impairments that clinical assessments register as disorder-specific symptom profiles.

The interplay between host genetic predisposition, environmental exposures, and the gut microbiome in the setting of autism spectrum disorder (ASD) are not fully understood. Although immunological and environmental mechanisms might play a role in ASD etiology, the evidence connecting specific copy number variations (CNVs), such as SHANK3, PTEN, MECP2, and others, to the changes in intestinal barrier function and the interrelationship between these genetic variations and gut microbiota is insufficient. This is why these relationships should be considered exploratory hypotheses for future studies investigating gene-specific mechanisms accounting for the associations between environmental factors, gut microbiome, and ASD.

### 4.4. Therapeutic implications and the path toward precision medicine

The most encouraging directions for microbiota directed treatment intervention include microbial metabolite signaling, intestinal barrier function, and immune regulation. Growing evidence from ASD probiotic and microbiota transfer studies [20],[21] plus preclinical murine studies [7],[22] offer some mechanistic and therapeutic findings for more microbiota related therapeutic investigations in ASD, although not conclusive evidence of efficacy. Conversely, there is currently limited data in ADHD to reach similar conclusions.

For these trials to succeed, the field must move beyond generic probiotic supplementation toward microbiome-informed patient stratification. Individuals with distinct baseline microbiome profiles, genetic risk architectures, or predominant mechanistic themes are unlikely to respond identically to the same intervention. Integration of microbiome profiling with host genomic data, immune phenotyping, and neuroimaging at study entry represents the methodological foundation for genuinely personalized microbiome medicine in NDDs.

### 4.5. Limitations and research priorities

Several limitations constrain conclusions from this synthesis. The predominance of cross-sectional and case-control designs means that directionality cannot be established: It remains theoretically possible that NDD behavioral phenotypes drive microbiome alterations rather than the reverse. Behavioral traits characteristic of ASD and ADHD, including food selectivity, restricted dietary repertoire, heightened stress reactivity, and reduced physical activity, may themselves reshape microbial composition, raising the important question of whether observed dysbiosis is a downstream consequence rather than an upstream driver of neurodevelopmental pathology. The bidirectional nature of the gut–brain axis means that both directions of effect may operate simultaneously, and disentangling primary from secondary dysbiosis will require prospective designs with pre-diagnostic microbiome sampling. Longitudinal studies beginning prenatally and tracking microbiome composition alongside neurodevelopmental assessments are essential. Second, heterogeneous 16S rRNA sequencing protocols, reference databases, and bioinformatic pipelines limit cross-study comparability. Standardized multi-omic protocols, combining metagenomics, metabolomics, and host transcriptomics, would substantially improve future research. Third, as a scoping review, this work maps mechanistic evidence breadth without formal quality or risk-of-bias assessment.

Future research priorities should include: Prospective birth cohort studies with serial microbiome sampling; mechanistic trials in genetically defined NDD subgroups; multi-omic studies resolving causal pathway architecture; and adequately powered clinical trials of specific probiotic and FMT protocols with standardized neurobehavioral, GI, and immune outcome measures.

## 5. Conclusions

In this scoping review, we provide a comprehensive overview of the important literature on the involvement of the gut microbiome and the gut–brain axis in ASD and ADHD, highlighting the variations in evidence between the two disorders. The outcomes for ASD include selected disorder-specific preclinical mechanistic literature, whereas the evidence base for ADHD is mainly correlational. Moreover, ASD is linked with disorder-specific preclinical mechanistic findings, while the evidence connecting ADHD and the microbiome is mostly correlational. The mechanisms by which the microbiome may affect dopaminergic or stress pathways in ADHD are therefore regarded as largely indirect and unsupported. The emerging intervention literature justifies cautious optimism that microbiome-directed strategies may complement existing therapeutic approaches, but realizing this potential requires longitudinal causal research, multi-omic analytical frameworks, and personalized intervention design. The gut–brain axis provides a theoretically supporter research model for developing understanding of neurodevelopmental disorders, but additional disorder-specific mechanistic and longitudinal findings are necessary before clinical utilization.

## Use of AI tools declaration

The authors declare they have not used artificial intelligence (AI) tools in the creation of this article.
